# The mutually reinforcing dynamics between pain and stress: mechanisms, impacts and management strategies

**DOI:** 10.3389/fpain.2024.1445280

**Published:** 2024-11-18

**Authors:** Nour Aboushaar, Natalia Serrano

**Affiliations:** ^1^ Global Medical Affairs, Bayer HealthCare LLC, Whippany, NJ, United States; ^2^ Ernest Mario School of Pharmacy, Rutgers, the State University of New Jersey, Piscataway, NJ, United States

**Keywords:** persistent pain, chronic pain, acute pain, pain management, stress

## Abstract

While distinct, pain and stress share complex biological and psychological mechanisms that—despite their protective functions—can lead to clinically maladaptive changes requiring therapeutic intervention when they recur or persist. Recognized as “worldwide epidemics” of modern life, both conditions significantly affect an individual's quality of life, functioning, and well-being; without timely intervention, they can become chronic, leading to substantial economic costs via healthcare expenses, lost wages, and reduced productivity. Evidence suggests that pain and stress not only feed into but exacerbate each other through a “vicious cycle,” driven by overlapping physiological, cognitive, and social mechanisms, indicating mutually reinforcing dynamics between pain and stress. In this review, we highlight the importance of recognizing the overlapping mechanisms that promote the persistence of pain and stress: (1) key physiological processes like maladaptive neuroplasticity, neuroendocrine dysfunction, and chronic inflammation; (2) cognitive and behavioral patterns such as fear avoidance, hypervigilance, and catastrophizing; along with (3) social, lifestyle, and environmental influences, such as socioeconomic status, lack of social support, and lifestyle choices. Through a case study, we illustrate the real-world implications of this vicious cycle perpetuating both conditions. We call for a paradigm shift in pain and stress management, advocating for a holistic management strategy encompassing pharmacological, psychological, and lifestyle interventions that address the underlying biopsychosocial factors. By fostering greater awareness among primary care practitioners and healthcare professionals, it is possible to better support individuals in breaking the cycle of pain and stress, thereby enhancing their quality of life and overall well-being.

## 1 Introduction

Both pain and stress are recognized as “worldwide epidemics” of modern life with serious implications for individuals and society. These states, while distinct, share complex overlapping biological and psychological mechanisms and commonly co-exist (1, 2). Despite having a protective function—signaling potential threats that demand an immediate response (e.g., to avoid injury)—recurrence or persistence of pain or stress responses can lead to long-term clinically maladaptive changes that offer no clinical benefit to the sufferer (3), and thus warrant therapeutic intervention.

In this review, we focus on the mutually reinforcing relationship between pain and stress and its impact on how individuals experience and perceive these states. We highlight the importance of recognizing the overlapping mechanisms that promote the persistence of pain and stress and illustrate how this establishes and perpetuates a “vicious cycle” that leads to reduced quality of life (QoL), functional limitation, and disability. We make the case that primary care practitioners (PCPs) and others who manage or counsel individuals presenting with pain should consider and address recurrent/episodic or persistent pain and chronic stress together. We also call for greater awareness of the possibilities of holistic and person-centered management approaches to help prevent or interrupt the vicious pain-stress cycle.

## 2 Basic concepts of pain and stress

Pain affects a significant proportion of individuals worldwide, with an estimated 27.5% of the world's population suffering from pain (9.9% to 50.3%) (4). Financially, pain incurs substantial costs, estimated at $560–$635 billion annually, due to healthcare costs, lower wages, and reduced work productivity (5).

The International Association for the Study of Pain (IASP) defines pain as “*an unpleasant sensory and emotional experience associated with, or resembling that associated with, actual or potential tissue damage*” (6, 7). Although this definition emphasizes the sensory and experiential aspects of pain as its core components, others favor a broader concept that also recognizes the cognitive and social aspects of pain as core features (6, 8). The “pain matrix” concept was proposed by Melzack and Casey (8) to integrate the sensory, emotional, and cognitive aspects of pain perception and processing. The pain matrix describes a network of brain structures that are collectively activated by painful stimuli. Garcia-Larrea and Peyron (9) subsequently expanded upon this, proposing a three-tier pain matrix that explicitly identifies (i) nociceptive (sensory), (ii) perceptive-attentional, and (iii) reappraisal-emotional mechanisms that shape the experience of pain. Nociceptive processing includes the initial detection and localization of pain, perceptive-attentional processing involves the brain's assessment and interpretation of these signals, and reappraisal-emotional processing encompasses the emotional response and memory formation related to the painful experience (9, 10). While highlighting the conceptual distinctions among the levels, Garcia-Larrea and Peyron also emphasize that, in practice, these levels and the brain regions involved are strongly interrelated (9). The brain regions involved in emotional and cognitive aspects of pain processing communicate with descending pain modulatory pathways in the spinal cord. Together, the brain and spinal cord are thus able to regulate (either inhibit or enhance) incoming pain signals through ascending and descending pain modulatory pathways (10–12). Concept definitions aside, it is widely accepted that a complex interplay of physiological, psychological, cognitive, behavioral, and social factors influence an individual's experience of pain and its impact on their health and well-being (8, 13).

Medically, various categorizations of pain (e.g., according to their origin/mechanism or duration) have been proposed to guide its diagnosis and treatment/management. Based on its origin, three major types of pain are currently recognized: nociceptive (related to tissue injury), neuropathic (related to nerve injury), and nociplastic (related to a “sensitized” nervous system). These are not mutually exclusive and often overlap or co-exist in individuals; nevertheless, the different underlying mechanisms need to be taken into account during pain assessment and management (1).

In terms of duration, distinctions have historically been made between “acute” and “chronic” pain (6, 7), but recent insights suggest that pain is better described as a spectrum (Table 1) (14). At one end is acute pain—often resulting from a specific injury or illness—which is short-lived, resolving within weeks (6, 7, 14). In “acute” settings, pain is understood as serving a protective role, alerting the individual to a threat or situation that requires a response to prevent harm and preserve well-being, and is expected to resolve upon removal of the painful stimulus or healing of the underlying injury (e.g., a wound or bone fracture). In recurrent/episodic pain conditions, the pain lasts for a relatively short time yet frequently reoccurs (14). Some examples include tension-type headaches and menstrual pain (Table 1). In some cases, acute pain may transition into a more persistent form of pain that lasts for several weeks and up to 3 months (14). This represents a critical phase for intervention to avoid progression to chronic pain. At the other end of the spectrum, chronic pain is identified in terms of its persistence for more than 3 months, often in the absence of or despite management of underlying causative factors (6, 7, 14). Within this framework, chronic pain is recognized as a disease state, and not merely a symptom, that warrants therapeutic intervention (7).

**Table 1:** Pain definitions in terms of duration and example conditions.

Pain category	Definition	Example condition(s)
Acute pain	Pain experiences and conditions that are typically short-lived and resolving within weeks. Such pain commonly arises from specific injuries, illnesses, or medical conditions, and usually subsides once the underlying issue is addressed (6, 7, 14)	Acute post-operative pain, pain in labor, fracture, and ulcer
Recurrent/episodic pain	Pain experiences and conditions that last for relatively short time yet frequently recurring over an extended period (14)	Migraine, polymyalgia rheumatica, dysmenorrhea, calcium phosphate deposition, sickle cell-associated pain
Transition from acute to Chronic pain	Pain experiences and conditions that last for a period ranging from several weeks to as long as 3 months (14)	Post-operative recovery
Chronic pain	Pain experiences and conditions which last longer than 3 months, often in the absence of or despite management of underlying causative factors (6, 7, 14)	Chronic low back pain, chronic postsurgical pain, chronic pelvic pain, and diabetic neuropathy

Stress can be understood in terms of physiological or psychological responses to daily life challenges (stressors). Pain, which activates physiological stress responses, is thus often considered alongside non-pain stressors within the stress literature. For many people, “stressful” or “distressing” situations are characterized by uncontrollable or unpredictable demands that are perceived as exceeding their ability to cope or control the source of stress. As with pain, whereas acute stress responses promote adaptation and enable individuals to maintain or restore homeostasis and stability, long-term or chronic stress is clinically maladaptive and has been associated with a greater risk of developing several chronic diseases (15, 16). In a survey commissioned by the American Psychological Association, 84% of American respondents reported having felt stressed in the previous 2 weeks. The top stressors reported were health-related (65%), work-related (64%), financial concerns (63%) and family responsibilities (55%) (17).

Just as people experience pain differently, they may perceive and react to stressors in significantly different ways. This variability may be related to individual factors, such as their perceived level of control and ability to handle the demands of the situation, and/or environmental factors like the level of social support they receive. Some individuals may view and respond to challenges positively, whereas others may experience overwhelming distress that impairs their coping mechanisms and leads to negative effects on behavior and physical health. Cumulatively, exposure to low-level stressors common in modern life (e.g., workplace stress, lack of sleep, environmental pollution) can overwhelm an individual's usual coping ability. This may lead to adoption of unhealthy coping mechanisms (e.g., overeating, social withdrawal, substance abuse) that can exacerbate the perceived stress and negatively impact overall well-being (15, 16).

There are notable parallels between pain and stress responses. Both enable organisms to react to immediate environmental threats/challenges and take action to promote their survival and well-being. However, clinically maladaptive changes can result in these states becoming recurrent or persistent. In the following sections, we discuss the relationship between pain and stress, focusing on several overlapping biopsychosocial mechanisms (physiological, psychological, emotional, cognitive, social, and behavioral).

## 3 What pain and stress have in common, and how they reinforce each other

Both pain and stress are complex conditions that often co-exist (18) and interact on multiple levels. Pain can strongly activate physiological stress responses (19); conversely, stress is known to be an important modulator of pain perception and responses (20). Under normal conditions, pain and stress responses are adaptive and protective processes. They maintain stability through physiological changes in response to physiological or psychological threats/challenges, a process termed allostasis (15, 21). With overuse and dysregulation of such processes in response to the cumulative burden of stressors (pain- or non-pain related) and life events, allostatic load (“wear-and-tear”) may occur (15, 22, 23). The balance between allostasis and allostatic overload, that is, the extent to which the body can successfully adapt or cope with stressors, is the net result of cost-benefit tradeoffs shaped by natural selection in populations (24). It has been posited that, although allostatic overload and chronic pain states are maladaptive clinically (at the individual level), these mechanisms may have had adaptive effects at the species or population level in the course of evolution (25). In modern human populations, on the other hand, allostatic overload is associated with clinically maladaptive pain and stress in individuals, with potential adverse impact on functioning and QoL.

### 3.1 Pain and stress: a mutually reinforcing relationship

Two models are widely used to conceptualize the relationship between pain and stress (20, 26, 27), which suggest mutually reinforcing dynamics. The first model seeks to explain the effect of pain in terms of its allostatic load. Since pain can activate physiological stress responses, repeated activation of stress responses by pain is thought to lead to “wear-and-tear” (allostatic overload) and progressive dysregulation of homeostasis (26). This can perpetuate a maladaptive state of persistent pain, interfere with normal activities and compromise well-being. The second model focuses on the “stress” side of the equation, proposing that prolonged or recurring stress, when perceived as unpredictable or unmanageable, can precipitate pain manifestations, such as headaches or musculoskeletal pain (27). Over time, this “wear-and-tear” can lead to maladaptive physiological and psychological responses that increase the individual's vulnerability to the effects of pain.

**Figure 1:**
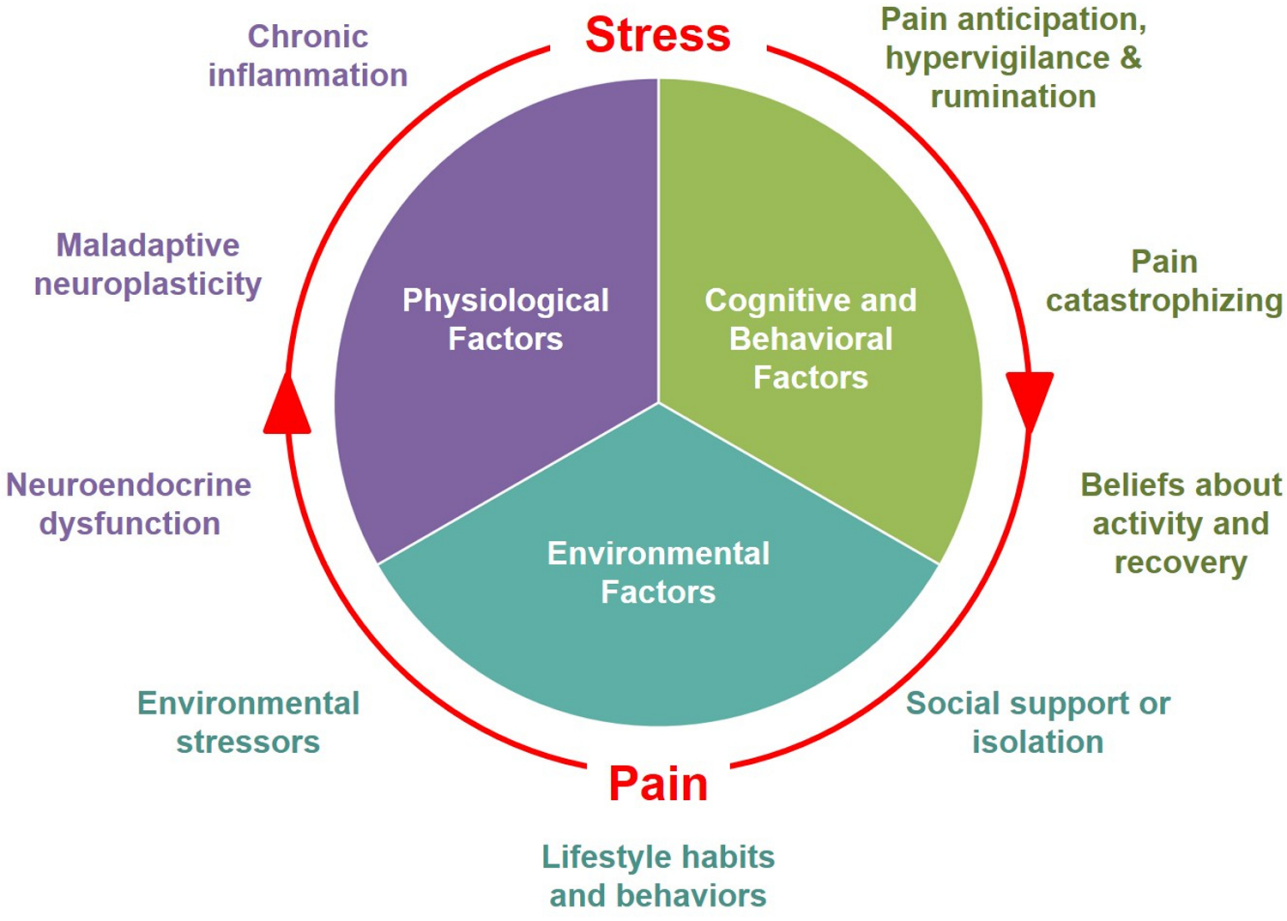
Biopsychosocial factors involved in the mutually reinforcing dynamics between pain and stress.

These models suggest that both recurrent or persistent pain and stress not only feed into but exacerbate each other, creating a “vicious cycle”. Figure 1 illustrates the mutually reinforcing dynamics between pain and stress, driven by the multiple overlapping biopsychosocial processes that contribute to both conditions. These mutually reinforcing effects can perpetuate a “vicious cycle” of interference with daily activities, reduced QoL, functional limitation, and disability. This cycle is sustained through the interaction of several maladaptive physiological mechanisms, cognitive responses, and social/environmental factors, which we discuss in the following sections.

### 3.2 Physiological mechanisms

Major physiological processes implicated in pain persistence include maladaptive neuroplasticity, neuroendocrine dysfunction, and chronic inflammation. Similar physiological changes/processes also occur in and can be exacerbated by chronic stress.

#### 3.2.1 Sensitization and maladaptive neuroplasticity

Amygdala plasticity and central sensitization are examples of maladaptive neuroplastic processes that can alter pain perception. These processes result in lasting structural changes in the central nervous system (CNS) or peripheral nervous system (PNS) that interfere with normal pain processing (28).

##### 3.2.1.1 Amygdala plasticity

The amygdala, situated in the limbic region of the brain, plays a major role in emotion- and mood-related aspects of pain perception (29). Within the amygdala, specialized neuronal circuits integrate and evaluate sensory-affective information in terms of pain (e.g., whether or not a stimulus is interpreted as painful, and the resulting emotional response). However, dysregulation in the balance of excitatory and inhibitory synaptic activity within the amygdala can occur with repeated exposure (pain-induced amygdala hyperactivity), leading to neuroplastic changes that persist beyond the initial pain stimulus (29). This remodeling and hyperactivity can promote pain persistence. Studies have demonstrated that exposure to acute and chronic stress can similarly lead to alterations in amygdala structure and function, resulting in amygdala hyperactivation (30, 31). Thus, the effects of stress on amygdala activity may also promote increased pain sensitivity or vulnerability to recurrent or persistent pain.

##### 3.2.1.2 Central sensitization

Central sensitization is a pathophysiologic process involving structural and functional alterations within the CNS that alter pain processing (32, 33). The presence of injury or inflammation within the nervous system can trigger heightened neural excitability and synaptic transmission, together with reduced inhibitory transmission in CNS neurons and circuits (33, 34). With central sensitization, the experience of pain becomes dissociated from the presence, duration, and intensity of the stimulus, resulting in allodynia (experiencing pain from previously non-painful stimuli) and hyperalgesia (amplified and sustained experience of pain from recognized painful stimuli) (33, 34). Examples of conditions in which central sensitization plays a role include fibromyalgia and migraine headaches, characterized by an increased sensitivity to pain that is often disproportionate to the external stimuli. These experiences can further heighten emotional distress, anxiety and pain catastrophizing (33). As for stress, this can also induce CNS plasticity through neuroinflammation, leading to a temporary hypersensitivity in sensory perception by the activation of descending pain modulatory pathways (35). The cumulative neuroplastic effects of pain and stress can therefore contribute to both amplification and persistence of pain.

#### 3.2.2 Neurotransmitter signaling and neuroendocrine dysregulation

##### 3.2.2.1 HPA axis exhaustion and cortisol dysfunction

Along with sympathetic nervous system activation by the amygdala, the hypothalamic-pituitary-adrenal (HPA) axis is a key pathway activated in response to pain and non-pain-related acute stressors. Upon HPA axis activation, corticotropin-releasing hormone, adrenocorticotropic hormone, and the “stress hormone” cortisol are released to regulate metabolism, inflammation, and other processes, temporarily enhancing the individual's ability to respond to the stressor (36). This is normally a transient response, with homeostatic mechanisms acting to halt the response once the stressor is removed (36).

Cortisol release enhances the body's ability to respond to stress but also contributes to fear conditioning, the formation of fear-based emotional memories that associate environmental cues with a stress response (37, 38). This conditioning has a sensitizing effect, causing environmental cues perceived as similar to reactivate these fear-based memories and trigger a stress response more readily. Frequent and repeated exposure to pain or non-pain-related stressors can lead to sustained HPA axis activation and increased cortisol secretion (37). Over time, repeated triggering of stress responses can lead to HPA axis exhaustion and hypocortisolism. Indeed, chronic stress-induced hypocortisolism has been described in various pain disorders, associated with heightened intensity of lower back pain and leg pain in individuals with chronic pain disorders, and emergence of new musculoskeletal pain (39–41).

##### 3.2.2.2 Aberrant pain processing through neurotransmitter imbalances: dopamine and serotonin

Dysregulation of pain modulation pathways can lead to aberrant pain processing. The rostral ventromedial medulla (RVM), which bridges the periaqueductal gray (PAG) matter and the spinal cord, plays a key role in descending pain modulation pathways. Within the RVM, there are neurons that respectively inhibit or facilitate pain signals (11, 12, 42), providing bi-directional control of pain perception. With recurrent episodes of pain, neurotransmitter imbalances can disrupt descending pain modulation, causing increased excitability and reduced pain signal inhibition (12). Over time, this dysregulation may play a role in the transition from acute to chronic pain.

The neurotransmitter dopamine mediates several brain functions including pleasure, reward, motivation, and motor control (43). Amplified pain perception and hyperalgesia have been linked to dysregulation of dopaminergic pathways, underscoring their role in modulating pain perception. Individuals in a prolonged hypodopaminergic state experience recurrent short bursts of dopamine secretion in response to pain, leading to aberrant learning of pain cues and increased vulnerability to the persistence of pain (44, 45). The presence of stress may compound the situation by reinforcing dopaminergic pathway dysregulation. One study showed that prolonged psychosocial stress impairs dopaminergic activity by reducing dopamine production capacity in the limbic striatum. This decline was associated with a mismatch in physiological and psychological responses to acute stress, characterized by suppression of stress-induced increase in blood pressure, alongside an intensified subjective stress response (46). These findings show how long-term exposure to psychosocial stress may lead to impaired dopaminergic activity and alter an individual's physiological and psychological responses to new stressors.

Serotonin is another important neurotransmitter with multiple functions, including emotion, cognition, inflammation and pain perception (47). Serotonin modulates pain perception through its effects on various neural pathways, including spinal descending excitatory or inhibitory pathways, and contributes to changes in synaptic transmission and neuroplasticity, which can affect pain perception and pain persistence (48). Depending on the intensity of the stimulus, type of receptor and brain region, serotonin may have nociceptive or antinociceptive effects (49). Besides pain perception, serotonin also modulates stress responses and is thought to be crucial in modulating the effects of acute and chronic stress (50, 51). The 5-HT1A and 5-HT1B receptors are important for both stress responses and pain transmission, and may serve to integrate these responses (52, 53). The serotonin pathway is also associated with pain-related anxiety, which can amplify pain perception and promote pain persistence (48).

Thus, both pain-related and non-pain-related stressors (such as adverse emotional and psychological impact) may contribute to the persistence of pain through their effects on neurotransmitter signaling and neuroplasticity.

#### 3.2.3 Chronic inflammation

Similar to pain, the effects of inflammation can be either protective or destructive. In the presence of tissue damage, an acute inflammatory response is initiated to combat internal threats and begin the repair and healing process. During inflammation, increased levels of pro-inflammatory mediators can trigger nociceptive activity in both the PNS and CNS, inducing the perception of pain. After the acute threat is resolved, homeostatic mechanisms then function to inhibit the inflammatory response. In contrast, persistent or chronic inflammation is maladaptive, occurring when acute inflammation fails to resolve. Prolonged inflammation in the PNS and CNS (neuroinflammation) is thought to contribute to the persistence of pain (54, 55).

Chronic inflammation, whether localized or systemic, has several adverse consequences. Localized chronic inflammation can lead to progressive tissue damage, contributing directly to pain persistence, whereas systemic inflammation may have more global and indirect effects on pain perception and experience. Systemic inflammation is associated with mood disorders including depression, anxiety and anhedonia, and chronic fatigue, due to the circulation of pro-inflammatory cytokines to the brain (56, 57). Psychosocial stress has also been found to promote both central and peripheral inflammation (58, 59). Thus, pain may persist if acute inflammation fails to resolve, and this may be further exacerbated in the presence of stress.

### 3.3 Cognitive and behavioral responses

When an individual perceives environmental demands as uncontrollable or unpredictable and believes these demands surpass their coping ability, both pain and non-pain stressors can trigger feelings of unease, rumination, and avoidance of stress-inducing stimuli (37). The fear-avoidance model describes this process, linking psychological factors, such as negative affectivity, negative appraisal, or anxiety, to an individual's perception of and/or reactions to pain (60). These psychological factors may manifest in maladaptive cognitive responses such as pain anticipation, catastrophizing, rumination, and hypervigilance, which can magnify or prolong the experience of pain. The adverse effects of many of these responses also appear to be exacerbated and/or prolonged in the presence of non-pain stressors, through overlapping physiological mechanisms activated by pain and stress, such as increased activity in the HPA axis and elevated cortisol secretion.

Pain catastrophizing refers to exaggerated negative responses to an actual or anticipated pain stimulus/threat, including magnified perception of pain, excessive rumination, and feelings of helplessness or inability to cope with pain. Catastrophizing is associated with altered connectivity in brain areas crucial for perceptual-attentional processing and emotional reappraisal, and other CNS regions involved in pain modulation (61). A key neurobiological mechanism by which catastrophizing influences pain circuitry involves prolonged HPA axis activation and increased cortisol secretion, which establish a primed physiological stress response that is easily activated (37). Catastrophizing correlates with worse pain-related outcomes (e.g., pain severity) and greater pain-related functional limitation and interference with activities (62).

Studies have also documented the neurobiological and perceptual impact of pain anticipation on the actual experience of pain. The perceived magnitude of pain can be influenced by expectations, e.g., anticipating a painful experience can influence the brain's response and increase sensitivity to a mild stimulus (63–65). Pain anticipation appears to enhance the functional connectivity between the anterior insula and the midcingulate cortex, which are both key components of the brain's salience network, even before the stimulus is presented. This increased connectivity facilitates the integration of information about the significance of an impending stimulus and influences an individual's perceptual decision-making (63).

Pain hypervigilance, which describes a state of excessive attention and sensitivity to pain and associated sensory input, has been linked to decreased pain thresholds and increased pain sensitivity in a range of pain-related disorders including fibromyalgia, osteoarthritis, and chronic back pain (66, 67). This hypervigilance may be generalized or directed towards specific areas of the body (e.g., the site of a past injury), and may also interact with central sensitization by strengthening facilitatory connections between the anterior cingulate cortex, involved in perceptual-attentional processes, and the PAG, a key component of the descending pain modulation pathway (34, 68).

Finally, maladaptive beliefs about rest or physical activity in relation to pain can lead to behaviors that enhance pain-related disability and reinforce the vicious cycle of pain and stress. Kinesiophobia (“fear of movement”) is a condition characterized by an overwhelming, irrational and incapacitating fear of engaging in physical activity, arising from the fear of experiencing pain and injury (69). A cross-sectional study investigating the relationship between psychosocial factors and pain revealed that patients with lower back pain who had kinesiophobia and similar beliefs were more likely to report and present with severe pain (70).

### 3.4 Social, lifestyle and environmental factors

Social, lifestyle, and environmental factors that can influence the experience and/or impact of pain include socioeconomic deprivation, lack of social support and isolation, obesity, physical inactivity, substance use (e.g., smoking or alcohol), and sleep disorders (1, 2).

#### 3.4.1 Socioeconomic, demographic and other factors

Socioeconomic deprivation and low socioeconomic (SES) status (e.g., income or education levels) are consistently found to be associated with higher prevalence of pain and greater pain-related disability (71–73). It has been suggested that SES-related stress (e.g., work/income instability, job insecurity, reduced social support) is a major contributing factor to these disparities (73). Other studies highlighted additional mechanisms: individuals with low SES were less likely to get their pain assessed and treated, and their pain experiences were often considered less legitimate, less severe and attributed to psychological factors, particularly when signs of emotional distress were present (74). Similarly, research has highlighted racial and ethnic health disparities, often intersecting with socioeconomic factors, in pain management outcomes as well as the experience of care. Such disparities have been described across acute and chronic pain settings, and include but are not limited to underdiagnosis, delayed treatment, and inadequate pain management (75–78).

As noted for other factors above, these relationships appear to be bi-directional. Persistent or recurrent episodes of pain can impair an individual's ability to perform their daily work-related and other roles. As such, it has consistently been linked to higher rates of absenteeism and lower work productivity, as well as heightened psychosocial stress and higher rates of job loss and/or economic inactivity (79–81).

#### 3.4.2 Poor social support and social isolation

Social support is proposed to improve health-related outcomes, including pain, by contributing directly to physical and psychological health, as well as indirectly by buffering or reducing the impact of stressors (77, 82). Studies in individuals with various chronic pain conditions indicate that social support is associated with a lower degree of pain severity and pain-related disability (13, 83). Conversely, pain can adversely affect social relationships, contributing to increased social isolation and reduced social support (84).

Although pain is a highly individual experience, it occurs within a wider social context, and can be strongly affected by others’ reactions to the way that the individual expresses their pain. The communal coping model (CCM) offers a useful framework for understanding how the social environment influences the individual's emotional and cognitive processing and their coping strategies in the presence of pain. The reactions of others, whether supportive/“positive” or critical/“negative”, can have a strong influence on cognition and emotional states, and thus affect pain perception. Interpersonal dynamics may thus indirectly influence outcomes, either by encouraging adaptive coping and helping to mitigate catastrophic cognitions, or by promoting maladaptive coping behaviors and exacerbating pain perception (85).

#### 3.4.3 Health-related lifestyle factors

Modern lifestyles are notably sedentary, in part due to profound transformations in work environments and leisure activity patterns (86, 87). A 2019 survey of 2,640 American adults revealed an average reported sedentary time of 9.5 h per day, including work and leisure time (86). Deskbound jobs and prolonged sitting in many workplaces put many individuals at risk of musculoskeletal pain, especially in the presence of poor posture, which is linked to musculoskeletal pain in the lower back, shoulders, knees, and thighs (87, 88). Pain-related fear-avoidance behaviors can lead to further reduced physical activity, aggravating the problem. This can be compounded by stress and/or anticipated stress, which have also been associated with reduced physical activity (89).

The modern-day diet, characterized by processed foods, added sugars, low nutritional diversity, and high caloric density, is another lifestyle factor significantly contributing to chronic pain development by increasing the prevalence of obesity and activating inflammatory pathways (90–92). The relationship between pain and obesity is well-established, with numerous studies demonstrating a robust positive association between the two (93). Notably, the relationship appears to be bi-directional: obesity is a risk factor for chronic pain development, and chronic pain in turn can promote obesity (93–97). The continued presence of pain is associated with functional limitation, lack of exercise due to pain avoidance, and disrupted sleep, all of which are obesogenic (93). Additionally, obesity is associated with a proinflammatory state; this, coupled with heightened mechanical stress on the body, further increases the likelihood of developing musculoskeletal and joint pain (98). Similarly, non-pain-related stress and obesity are linked through various metabolic, neurobiological, and behavioral processes. Chronic stress can promote obesity by persistently activating the HPA axis, triggering changes in brain reward processing, as well as the gut microbiome. Additionally, it may contribute to obesogenic behavior such as overeating and “emotional eating”, consumption of highly-processed “junk” foods, reduced physical activity, and sleep disturbance (99, 100). Together, these factors can create a cycle that reinforces and exacerbates the impact of stress/pain on the individual.

Stress associated with persistent or recurring pain could lead some individuals to substance use (e.g., smoking, or excessive alcohol consumption) as a coping mechanism. Heavy smoking is associated with more intense pain and greater impact of pain, including worsened physical functioning and sleep, increased tiredness, and higher rates of depression, anxiety, and anger (2, 101–104). Smokers with chronic pain had poorer prognosis with respect to pain management and recovery (104). Studies in individuals who use alcohol as a coping mechanism suggest that excessive alcohol consumption may actually be associated with greater pain severity (105). For example, a study of community-dwelling older adults who used alcohol to manage pain reported a higher prevalence of reported moderate-to-severe pain in problem drinkers than in non-problem drinkers (106). Excessive alcohol consumption is also associated with other health conditions such as alcohol-induced pancreatitis, neuropathy, or osteoarthritis, that may increase the individual's pain burden (105).

Pain and sleep disturbance have a bi-directional relationship mediated through complex biological and psychological mechanisms, similar to that observed with obesity (107, 108). Chronic sleep deprivation or poor sleep quality have negative effects on health and well-being, and can adversely affect an individual's pain perception or experience (107, 109). For example, poor sleep elevates inflammatory markers, contributes to HPA axis dysregulation, and alters neurotransmitter levels affecting pain thresholds, resulting in increased pain sensitivity (107, 108). It is also well known that individuals with poor sleep quality exhibit reduced resilience to stressors (110, 111), which may contribute to chronic stress. Poor sleep has also been shown to be a risk factor for a range of adverse health outcomes, including disability due to pain-related conditions (107–109).

In conclusion, the relationship between pain and stress can be described as mutual reinforcement, rooted in several overlapping processes that contribute to both conditions. As described above, key physiological processes include neuroplastic remodeling and central sensitization, neuroendocrine dysfunction, and chronic inflammation. Cognitive-behavioral responses that contribute to the mutually reinforcing relationship between pain and stress include fear conditioning and avoidance, pain hypervigilance, anticipation, and catastrophizing, as well as exaggerated negative appraisal and distorted beliefs about pain, activity, and recovery (Figure 1).

### 3.5 Impact of pain and stress dynamics on quality of life

Together, pain and stress can have substantial adverse impact on an individual's physical and psychological health, and on their overall QoL (112, 113). Studies have documented the numerous adverse effects of persistent pain on QoL: impaired physical functioning, mood dysregulation, sleep disturbance, impact on social relationships and professional life, and diminished overall life satisfaction (114–117).

Similarly, studies in different populations suggest that the presence of excessive or chronic stress can have a significant negative impact on QoL and self-reported health (118–120). Common reported stressors include socioeconomic factors (e.g., financial worries, job security, social relationships, daily life pressures) and personal factors (e.g., health concerns, family life), with health concerns, family life and daily life pressures exhibiting strong associations with poor QoL (119).

### 3.6 Case study

**Figure 2:**
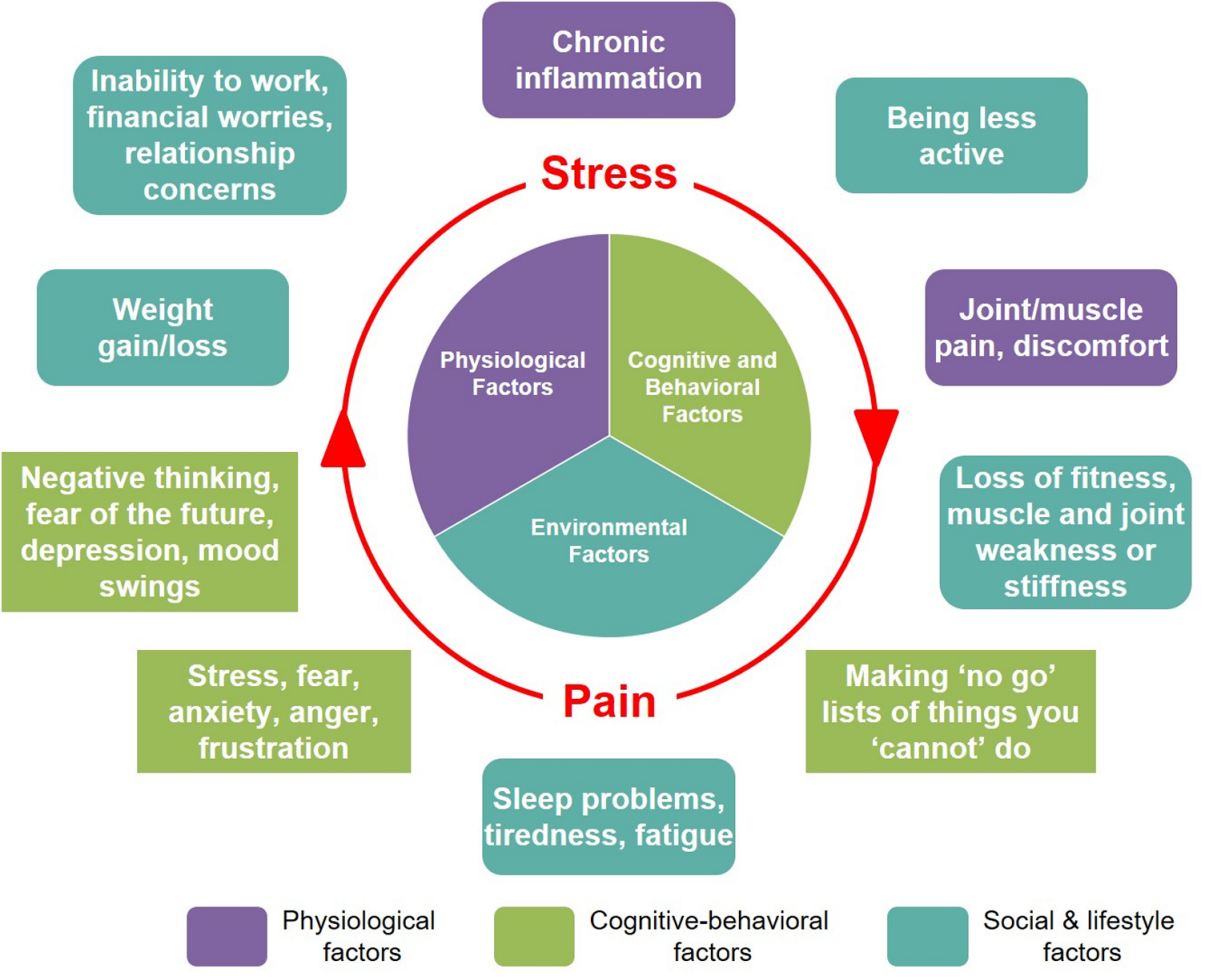
This hypothetical case study illustrates the potential for the cumulative effects of exposure to multiple physical and psychological stressors, including pain, to establish and reinforce a state of persistent pain and stress.

We explore the impact of mutually reinforcing pain and stress (Figure 2) through a hypothetical case study of a 31-year-old female marketing professional. With a typical modern lifestyle, this individual balances the demands of her career (tight deadlines, long working hours, excessive computer use) with her personal goals and social engagements. Although not diagnosed with any chronic illnesses, she is overweight and does not have a healthy lifestyle, with very limited physical activity outside of work, a diet consisting mostly of convenience foods, and irregular sleeping habits. For the last 1 year, she has suffered recurrent tension-type headaches and pain in her back, neck, and shoulders, which she attributes to extended periods of sedentary desk work. Fearing that any vigorous physical movement will make her muscle/joint pain worse, she puts off engaging in any physical exercise. The intermittent but severe headaches she experiences significantly disrupt her work and sleep. She expresses worry and frustration about feeling “unhealthy”, inability to cope with the demands of her work, and fears about losing her job due to her reduced productivity. She reports considerable anxiety and stress related to her workplace and supervisors, whom she feels are unsupportive*.* She mentions frequently needing to use painkillers, and also spending much of her personal time “scrolling mindlessly” on social media or watching television to distract herself from the stress she is experiencing. After her most recent headache episode, she experienced mood swings, leading to conflict at home and further increasing her feelings of stress: “*And then the pain gets worse… I can't make it go away*”. She went to her doctor to ask for “*painkillers that are strong enough*” to solve her problems. Her doctor explained the complex, individual, and context-dependent aspects of how pain is perceived and experienced, emphasizing the need for a holistic approach to help her address what she is experiencing. Her recommended treatment plan combined pharmacological treatment [over-the-counter (OTC) analgesics with anti-inflammatory activity], with non-pharmacological strategies. These included changes to improve her diet and sleep habits, suggestions for ergonomic improvements at work, and exploring stress-reduction practices such as mindfulness and yoga.

## 4 Holistic and person-centered management of pain and stress

With the complex interplay of factors that contribute to the vicious cycle of pain and stress, it is crucial to consider holistic and coordinated person-centered approaches to managing both. Such approaches can and should encompass biomedical, psychological, social, and environmental/lifestyle interventions to address the range of factors that contribute to chronic stress and persistence of pain.

As a starting point, adequate attention to assessing and treating pain, including its potential underlying causes, is crucial. Besides understanding the heterogeneity of pain types and underlying causes, care providers also need to keep in mind the complex, contextual, and individual nature of pain perception and experience. This can help in identifying at-risk individuals with clinical or psychosocial factors (e.g., possible central sensitization, catastrophizing traits) that may hinder recovery as expected, or those at risk of developing persistent pain. The “yellow flag” system originally proposed by Kendall for psychosocial assessment of patients with chronic pain (121) helps identify psychological and other (e.g., work-related, systemic/environmental) risk factors that may be important to investigate and address, or situations where referral is indicated. Both chronic pain and stress are known to have close connections to a number of mental health conditions, such as anxiety, depression, or substance use (10). Care providers should be aware of these connections, and should ideally be able to identify and refer individuals appropriately, if they are not equipped with the necessary knowledge/skills to manage these comorbid conditions.

Although OTC analgesics (e.g., ibuprofen, aspirin, acetaminophen) are widely available and generally effective for relieving most types of acute pain (122), pain that persists could be a sign of more serious underlying medical or psychiatric issues. It is therefore important for primary care providers to carefully assess the individual's pain-related and non-pain-related symptoms and overall condition, and advise/counsel them accordingly. This includes the possibility of referrals for specialist assessment; some examples are individuals who may meet clinical diagnostic criteria for anxiety or depression, sleep disorders, or those with complex pain conditions that require specialist management.

Successful management may involve multimodal interventions (biomedical, psychosocial, environmental) that target either or both pain and stress together (Table 2). In addition to standard pharmacological (OTC or prescription medications) and medical therapies, complementary and alternative medicine interventions such as acupuncture or massage therapy have also shown promise for relieving pain and supporting recovery. A meta-analysis concluded that there is evidence for the efficacy of acupuncture for pain relief (123) and of massage therapy for stress and pain relief, likely mediated by cortisol reduction and increased release of both serotonin and dopamine (124).

**Table 2:** Approaches for managing pain and stress.

Domain	Modalities & strategies
Physiological	Pharmacological interventions including analgesics or agents with anti-inflammatory or anxiolytic effects
	Medical interventions including corticosteroid injections, nerve blocks, transcutaneous electrical nerve stimulation
	Complementary/alternative medicine such as massage, acupuncture, natural products
Cognitive and behavioral	Psychological interventions including cognitive behavioral therapy, acceptance and commitment therapy, mindfulness-based interventions, biofeedback
	Mind-body techniques—yoga, Tai Chi, meditation, relaxation therapies
	Pain education
	Stress management
Social, lifestyle and environmental	Lifestyle/behavioral interventions addressing diet, weight management, physical activity, sleep, stress
	Peer support groups

Cognitive factors should also be considered in determining the appropriate management approach. As discussed in earlier sections, an individual's pain-related thoughts, emotions and behaviors can significantly influence their recovery process and outcomes (8). Person-centered psychological interventions such as cognitive behavioral therapy (CBT), acceptance and commitment therapy (ACT), and mindfulness-based interventions (MBIs), may help the individual to manage their pain-related perceptions and maladaptive responses, and allow them to establish or persist with healthier coping strategies (1, 44). CBT focuses on reshaping the maladaptive beliefs, attitudes, and behaviors that exacerbate the burden of disease. Neuroimaging studies have shown that CBT can increase engagement of key brain areas involved in pain modulation and reappraisal, such as ventrolateral prefrontal/lateral orbitofrontal cortex, in patients with chronic pain conditions (125). By enhancing psychological flexibility, ACT can help the individual to reshape their relationship and reactions to pain, encouraging increased acceptance and continued participation in life activities despite the continued presence of pain (10, 126). ACT has been shown to improve chronic pain-related outcomes (127), and neuroimaging studies indicate that ACT leads to increased brain activity across essential networks involved in self-reflection, emotion, and cognitive control, such as default mode network, the frontoparietal network, and the salience network (126). MBIs may help individuals deal with stress and pain by improving their awareness of and ability to regulate their attention, cognitions, and emotions in the presence of painful or stressful experiences. Neuroimaging studies suggest that MBIs can modulate pain perception through effects on brain regions, such as thalamus, prefrontal and somatosensory cortical regions, that are involved in sensory processing, attentional redirection, and emotional reappraisal (128, 129).

Other promising approaches include biofeedback therapy, which utilizes techniques such as deep breathing and progressive muscle relaxation (130, 131). These techniques have been found to have a positive impact on stress levels (132). Along with developing greater awareness and coping skills, pain science education can be useful to help individuals deal with cognitive issues or behaviors that negatively influence their experience of pain (133). Mind-body practices such as tai chi and yoga have gained attention as beneficial strategies for pain and stress management (134, 135). Physical therapy, in tandem with stress management, can also be valuable in pain rehabilitation (37, 136).

Lifestyle interventions directed at improving physical activity (137–139), diet (92) and sleep (140, 141) can promote better overall health, and are likely to also enhance resilience and coping with pain and/or stress. Finally, the presence of strong social support, whether from friends and family or peer groups, is beneficial for physical and psychological health, and has been linked to greater resilience to stress (142).

## 5 Conclusion

Due to a range of common underlying biopsychosocial factors, recurring or persistent stress and pain can mutually reinforce and exacerbate one another. Both conditions can significantly impact an individual's QoL, functioning, and well-being, and without timely intervention, may develop into long-term or chronic conditions. With this complexity, many individuals will benefit from a comprehensive person-centered management approach that may encompass pharmacological and non-pharmacological approaches. We advocate for greater awareness among primary care practitioners and healthcare professionals to recognize and mitigate the effects of these “worldwide epidemics” by treating the person, not only their pain.
